# Combination of Trans-Resveratrol and ε-Viniferin Induces a Hepatoprotective Effect in Rats with Severe Acute Liver Failure via Reduction of Oxidative Stress and MMP-9 Expression

**DOI:** 10.3390/nu13113677

**Published:** 2021-10-20

**Authors:** João C. Fernandes, Elizângela G. Schemitt, Juliana Da Silva, Norma P. Marroni, Ana Lima, Ricardo B. Ferreira

**Affiliations:** 1 Linking Landscape, Environment, Agriculture and Food (LEAF), Universidade de Lisboa, Instituto Superior de Agronomia, Tapada da Ajuda, 1349-017 Lisbon, Portugal; agusmaolima@gmail.com (A.L.); rbferreira@isa.utl.pt (R.B.F.); 2 Laboratory of Experimental Hepatology and Gastroenterology, Hospital de Clínicas de Porto Alegre, Porto Alegre 90040-060, Brazil; elizschemitt@gmail.com (E.G.S.); nmarroni@terra.com.br (N.P.M.); 3 Laboratório de Genética Toxicológica, Universidade Luterana do Brasil, Canoas 92425-900, Brazil; juliana.silva@ulbra.br; 4 Faculty of Veterinary Medicine, Universidade Lusófona de Humanidades e Tecnologias, Campo Grande 376, 1749-024 Lisbon, Portugal

**Keywords:** inflammation, liver failure, MMP-9, oxidative stress, resveratrol, stilbenes, thioacetamide, ε-viniferin

## Abstract

Stilbenes are a major grapevine class of phenolic compounds, known for their biological activities, including anti-inflammatory and antioxidant, but never studied in combination. We aimed to evaluate the effect of trans-resveratrol + ε-viniferin as an antioxidant mixture and its role in inflammatory development an in vivo model of severe acute liver failure induced with TAA. Trans-resveratrol + trans-ε-viniferin (5 mg/kg each) was administered to Wistar rats. Resveratrol + ε-viniferin significantly decreased TBARS and SOD activity and restored CAT and GST activities in the treated group. This stilbene combination reduced the expression of TNFα, iNOS, and COX-2, and inhibited MMP-9. The combination of resveratrol + ε-viniferin had a hepatoprotective effect, reducing DNA damage, exhibiting a protective role on the antioxidant pathway by altering SOD, CAT, and GST activities; by downregulating TNFα, COX-2, and iNOS; and upregulating IL-10. Our results suggested that adding viniferin to resveratrol may be more effective in hepatoprotection than resveratrol alone, opening a new perspective on using this stilbene combination in functional diets.

## 1. Introduction

**Figure 1:**
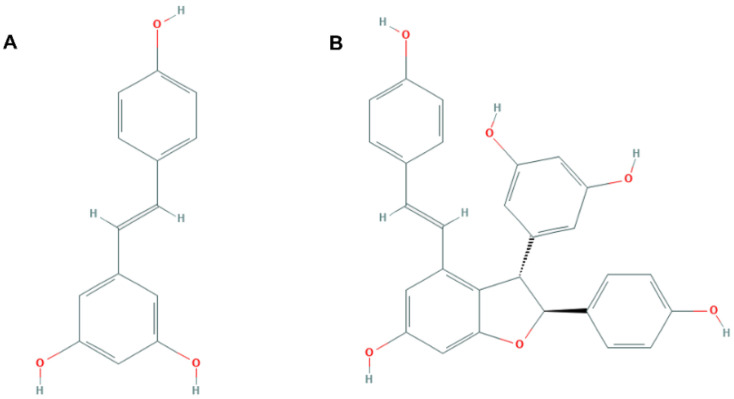
The chemical structures of trans-resveratrol (CID: 445154) (**A**) and trans-ε-viniferin (CDI: 5281728) (**B**).

The search for possible plant metabolites to develop novel food components with improved health-promoting properties is gaining interest among both the scientific community and the food industry [1]. Grapevine (*Vitis vinifera*) is one of the most used plants worldwide, and is a repository of bioactive compounds. Stilbenes are a major class of phenolic compounds present in grapevine. Trans-piceid, trans-resveratrol, ε-viniferin, and trans-pterostilbene have all been reported in grapes [2]. Trans-resveratrol (Figure 1A) and other stilbenes have been extensively investigated for their biological activities, including anticancer, anti-inflammatory, and antioxidant activities [3], and including in liver damage. Indeed, it has been established that moderate wine consumption is associated with some health benefits derived from the presence of stilbenes, including hepatoprotective activities. Emerging in vitro and in vivo studies corroborate that trans-resveratrol exhibits therapeutic effects on some liver disorders, leading to, for example, a significant increase in survival rates after liver transplantation, as well as a decrease in fat deposition, necrosis, and apoptosis. Coincidentally, it also provides liver protection against chemical, cholestatic, and alcohol injuries. Trans-resveratrol can also decrease liver fibrosis and steatosis. On the other hand, some studies showed that viniferins might have stronger antioxidant activities than trans-resveratrol, and could inhibit reactive oxygen species production [4], but they have been far more neglected in these studies. On this matter, some isolated stilbenes have been investigated for their ability to cancel or mitigate oxidative stress and inflammation in hepatic diseases. Trans-ε-viniferin (Figure 1B) is a dehydrodimer of trans-resveratrol formed from resveratrol by oxidation [5]. This natural stilbenoid is synthesized by *V. vinifera* in response to biotic and abiotic stresses, and can be constitutively found in all grapevine woody parts. Trans-ε-viniferin may provide superior therapeutic properties than those observed with trans-resveratrol, but may also be more toxic. The antiproliferative and proapoptotic effects of trans-resveratrol and its oligomers, including ε-viniferin, on HepG2 cancer cells and human colon cancer cells have also been previously suggested with isolated compounds [5].

Severe acute liver failure (SALF) is a unique and seldom-observed syndrome of severe injury to liver cells without any pre-existing liver disease. It manifests unexpectedly with a rapid onset and is frequently fatal, affecting previously healthy individuals [6]. SALF is characterized by a set of inflammatory processes that can lead to parenchymal damage, which may evolve to fibrosis, and in some cases liver cancer, in patients without known liver disorders [7]. One of the main causes of SALF is hepatotoxicity caused by chemical agents, which damage hepatocytes and cause an increase in reactive oxygen species (ROS). The hepatotoxic metabolites bind to a toll-like receptor 4 (TLR4) complex that triggers the transcription of nuclear factor kappa B (NFκB), activating the production of proinflammatory cytokines, such as tumor necrosis factor-α (TNFα) and interleukin-6 (IL-6); and modulation of IL-10, an anti-inflammatory cytokine [8,9]. Additionally, it can increase the production of the enzymes cyclooxygenase-2 (COX-2) and nitric oxide synthase (iNOS), both of which act as inflammatory mediators [10]. Another family of enzymes involved in inflammatory diseases are the matrix metalloproteinases (MMPs). MMPs are zinc-dependent endopeptidases, which are key participants in the remodeling of the extracellular matrix (ECM) under physiological and pathologic conditions. Although MMPs’ antifibrotic effects, due to their ability to degrade ECM proteins, are well established, they can also exert profibrotic effects under various pathological conditions [11], including hepatocellular carcinoma (HCC) [12]. MMP-9 seems to play a major role in tumor angiogenesis, and when overexpressed in HCC, a higher tumor progression is noted due to increased lymph node invasion, promotion of metastasis, deficient differentiation, and overall poor prognosis [13]. Liver injury and regeneration have also been correlated to complex ECM-related pathways, and several works [14] have suggested that MMP-9, and particularly MMP-2 (generally known as gelatinases), fulfill important roles in liver inflammation, fibrosis, and cancer, with enough studies justifying the rationale to search for inhibitors to specifically target MMP-2 and -9 in liver.

The therapies to overcome SALF include intensive and general use of drugs, but the only effective treatment to date is liver transplantation [15]. The availability of organs is limited, so the use of alternative therapeutic applications, such as plant biocompounds, is extremely important. Due to their potential protective role for liver and inflammatory diseases, stilbenes may be excellent candidates for novel therapeutic approaches supported by preventive diets. Although they are usually approached as isolated compounds, a recent study by Mattio et al. [16] showed that higher pancreatic α-amylase inhibition was observed when resveratrol dimers were mixed. Since trans-resveratrol shows high hepatoprotective activities, it seems promising that the viniferins may be even more effective than trans-resveratrol as a potential therapeutic approach to liver injury. Nonetheless, these compounds have never been studied together. Since they are indeed present in grapevines, this type of approach could open novel insights on the functional bioactivity of grapevine-derived foods. Furthermore, the usage of vitis byproducts is also a way to address the disposal problems arising from the considerable amounts of residues that the wine and grapevine industry generate, and in this way, incorporate them in novel foods, increasing sustainability and the circular economy as well.

Within this context, the present study aimed to evaluate the effect of a combination of trans-resveratrol and ε-viniferin on the oxidative stress and inflammatory process in an in vivo model of SALF induced by thioacetamide (TAA), and infer its nutraceutical or dietary potential to liver injury.

## 2. Materials and Methods

### 2.1. Animal Experimental Procedures

Twenty-eight male Wistar rats (weight ± 300 g) were selected for this study. Animals were housed in boxes (47 cm × 34 cm × 18 cm) lined with wood shavings, in a cycle of 12 h light/dark, and maintained at a temperature of 18 to 22 °C. Water and food were given ad libitum. The animals were randomized into four groups (*n* = 7): control (CO), control plus trans-resveratrol + trans-ε-viniferin (CO + RV), thioacetamide (TAA), and TAA plus trans-resveratrol + trans-ε-viniferin (TAA + RV).

Severe acute liver failure (SALT) was induced by TAA with the administration of two intraperitoneal doses of 400 mg/kg in normal saline solution (0.9% *w*/*v* NaCl), with an 8 h interval between doses [17]. The animals in the control groups (CO) received 0.9% saline solution (5 mL). Resveratrol + ε-viniferin-treated groups received intraperitoneally a mixture of trans-resveratrol and trans-ε-viniferin, 5 mg/kg each in 1 mL 0.9% (*w*/*v*) NaCl. Then, 30 min after the last dose of TAA, the first dose of RV was delivered; the second and third doses of RV were administered 24 and 36 h, respectively, after the start of the experiment (Supplementary Material Figure S1). The resveratrol used in this experiment was purchased from TargetMol (Ref.: T1558; purity 99.90%), and the ε-viniferina from Actichem (Ref.: Vini 138-18; purity > 85%, purified by HPLC).

The animals were weighed and sedated using a mixture of ketamine hydrochloride (95 mg/kg) and 2% (*v*/*v*) xylazine hydrochloride (8 mg/kg) administered by intraperitoneal injection 48 h after the start of the experiment. The abdominal region was shaved and disinfected, and a midline ventral laparotomy was performed. Blood was then collected from the heart and placed into a test tube containing heparin to prevent coagulation.

After collecting blood, animals received an overdose of anesthetic (three times the therapeutic dose, according to the CONCEA guidelines). After death confirmation, the liver was removed in sections for storage and subsequent analysis.

Animal handling followed the ethical principles for animal experimentation mandated by current Brazilian legislation (Law No. 11794/2008) [18], the standards of the Brazilian Council for the Control of Animal Experimentation (CONCEA) [19], the State Code for Animal Protection, and established local procedures for the care and use of animals in experimental research.

### 2.2. Spectrophotometric Analysis of Biochemical Parameters

Liver homogenates’ protein content was determined by the Bradford method [20]. Lipid peroxidation was assessed by the thiobarbituric acid-reactive substances (TBARS) method [21]. Levels of the catalytic activities of superoxide dismutase (SOD) were evaluated in rat liver extracts [22], as well as of the antioxidant enzyme catalase (CAT) [23] and the phase-II detoxification enzyme glutathione S-transferase (GST) [24].

### 2.3. Multiplex Analysis

Hepatic tissue homogenates were used to measure the levels of IL-6 and IL-10 using a MILLIPLEX™ MAP Rat Cytokine bead-based multiplex assay kit (RCYTO-80K, Millipore, Billerica, MA, USA), in accordance with the manufacturer’s recommendations. A 1:5 dilution of tissue specimens was incubated in duplicate overnight with IL-6 or IL-10 capture beads. The beads were then washed and incubated for 2 h with the biotin-conjugated detection antibody, and subsequently for 30 min with streptavidin–phycoerythrin. A Luminex 100 IS Multiplex BioAssay analyzer (Luminex Corporation, Austin, TX, USA) was used to read the bead fluorescence. Cytokine concentrations were determined from these readings using four standard curves. Results were expressed as pg/mL.

### 2.4. Gene Expression Analyses

RNA rat liver samples were extracted using the NZY Total RNA Isolation kit (NZYTech, Lisboa, Portugal) according to the manufacturer’s protocol. Reverse transcription was carried out using the RevertAid reverse transcriptase priming with the oligo-d(T) kit (Thermo Scientific, Waltham, MA, USA) according to the manufacturer’s suggestions.

Quantitative real-time PCR was used to analyze known inflammation-related genes (IL-6, IL-10, MMP-9, COX-2, iNOS, TNFα, and NFκB), using an iQ5 Real-Time PCR (BioRad, Hercules, CA, USA), and was performed in 20 μL reaction volumes comprising cDNA resulting from 2 µg RNA, 0.5 μM gene-specific primers (Supplementary Material Table S1) in SsoFast™ EvaGreen^®^ Supermixes (Bio-Rad, Hercules, CA, USA).

The cycling conditions were: 95 °C for 3 min followed by 40 cycles of 95 °C for 10 s, 61 °C for 25 s, and 72 °C for 30 s. In each case, melting curves were generated in order to confirm a single product’s amplification and the absence of primer dimerization. Each analysis was performed in triplicate biological reactions. The corresponding quantification cycles (Cq) were obtained using the iQ5 optical system software (Bio-Rad, Hercules, CA, USA) and exported to an MS Excel spreadsheet (Microsoft Inc., Redmond, WA, USA) for further analysis. Cq values of each gene of interest were normalized with respect to glyceraldehyde 3-phosphate dehydrogenase (GAPDH). Relative gene expression values were represented as fold-change values in relation to the control conditions.

### 2.5. Alkaline Comet Assay

The comet assay was carried out according to Tice et al. [25] with minor modifications [26]. Blood samples (50 µL) were transferred to heparin solution (Liquemine^®^ 25000 UI, 10 µL, Roche, Basel, Switzerland). Cell suspensions (5 µL) were embedded in 95 µL of low-melting-point agarose 0.75% (*w*/*v*; GibcoBRL) and spread onto already-prelayered agarose microscope slides. The slides were gently washed with PBS and then placed in cold lysis buffer (2.5 M NaCl, 100 mM EDTA, Trizma base 10 mM, freshly added Triton X-100 1% *v*/*v* (Sigma Aldrich, Burlington, MA, United States) and DMSO 10% *v*/*v*, pH 10) until 48 h at 4 °C. After this, the slides were incubated in recently prepared alkaline buffer (300 mM NaOH and 1 M EDTA, pH > 13) for 20 min at 4 °C in an electrophoresis cube. An electric current of 300 mA at 25 V (0.90 V/cm) was applied for 15 min. Subsequently, a neutralized step was carried out using 0.4 M Tris-HCL (pH 7.5), silver stained, and examined under a microscope. Then, 100 randomly selected images of nucleoids (50 nucleoids from each slide) from each animal were observed. Nucleoids were also visually scored according to tail size into five groups, varying from undamaged (0) to maximally damaged (4), resulting in a DNA damage score for each animal, and therefore for each studied group. The damage index (DI) ranged from 0 (completely undamaged, 100 cells × 0) to 400 (maximum damage, 100 × 4). The damage frequency (DF) calculation was based on the number of nucleoids with tail versus those with no tail.

### 2.6. Statistical Analysis

All data are presented as mean values ± standard deviation (SD) of an appropriate number of replicates in each experiment. The results were statistically assessed by variance analysis (ANOVA) and post hoc Student–Newman–Keuls test with *p* < 0.05 to compare the significance of each treatment. SigmaPlot (Systat Software Inc., San Jose, CA, USA) was used for statistical analysis.

## 3. Results

The effect of a mixture of trans-resveratrol and trans-ε-viniferin on thioacetamide (TAA)-induced severe acute liver failure (SALT) was assessed in rats. Twenty-eight rats were assembled into four groups of seven animals each: (1) the control group (CO), (2) the CO + RV group (which received intraperitoneally a mixture of trans-resveratrol and trans-ε-viniferin), (3) the TAA group (which received an intraperitoneal administration of TAA to induce SALT, and (4) the TAA + RV group (which received intraperitoneally both TAA and the mixture of trans-resveratrol and trans-ε-viniferin). The timings, concentrations, and volumes of the administrations are given in the Materials and Methods section.

### 3.1. Assessment of Oxidative Stress

**Figure 2:**
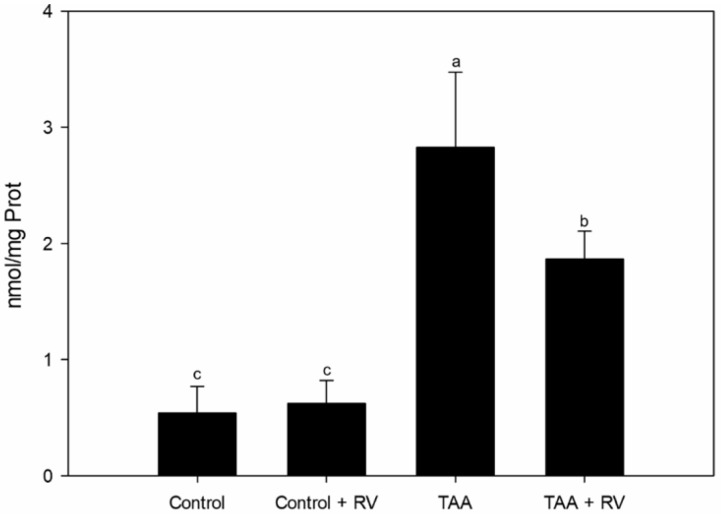
Lipid peroxidation assessment in the liver of rats with severe acute liver failure. Values are expressed as the mean ± standard deviation. Lowercase letters indicate significant differences between treatments (*p* < 0.05), *n* = 7. RV: resveratrol + ε-viniferin; TAA: thioacetamide.

Lipid peroxidation evaluation by the TBARS method (Figure 2) unveiled a significant reduction in lipid peroxidation in the TAA + RV group, to which resveratrol and ε-viniferin were administered, as compared with the TAA group (*p* < 0.05).

Organisms rely on an antioxidant defense system against ROS, which involves a number of enzymes such as superoxide dismutase (SOD), catalase (CAT) and glutathione S-transferase (GST).

**Figure 3:**
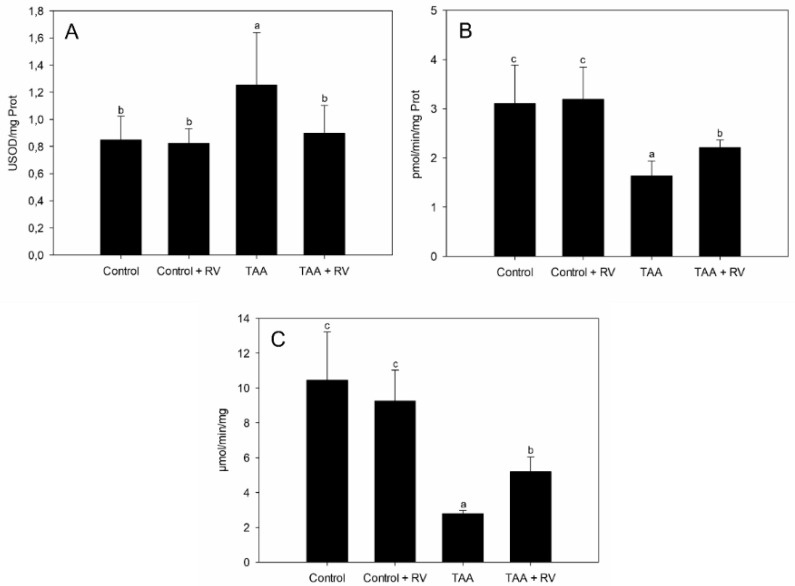
Effects of resveratrol and ε-viniferin on the activity of catalase, glutathione peroxidase, and glutathione S-transferase in the liver of rats with experimental severe acute liver failure: (**A**) SOD, (**B**) CAT, and (**C**) GST. Values are expressed as the mean ± standard deviation. Lowercase letters indicate significant differences between treatments (*p* < 0.05), *n* = 7. RV: resveratrol + ε-viniferin; TAA: thioacetamide.

Figure 3 shows the results obtained from the four groups of rats concerning the liver levels of catalytic activities of the antioxidant enzymes CAT and SOD, and of the detoxifying enzyme GST. In the TAA group, SOD activity increased in comparison to the CO groups (*p* < 0.05), whereas the additional administration of ε-viniferin and resveratrol decreased the SOD levels to a level similar to controls (*p* < 0.05). CAT and GST activities were significantly reduced in the TAA groups as compared to controls. However, this reduction was particularly intense in the livers of the untreated, SALT-affected rats, with the additional administration of resveratrol and ε-viniferin reversing results that were intermediate between the normal liver and the TAA-affected liver.

### 3.2. Assessment of the Inflammatory Process

**Figure 4:**
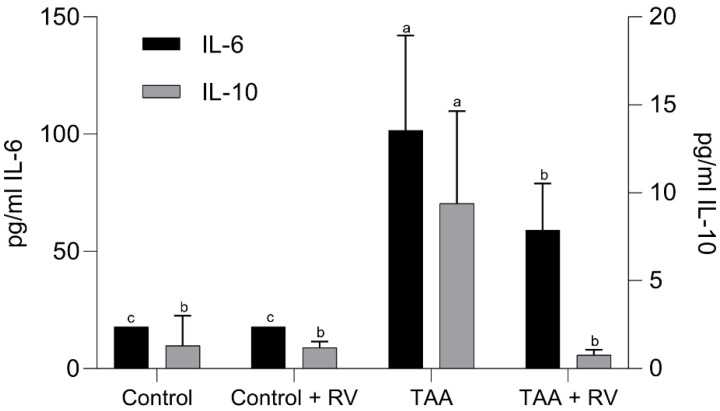
Effect of resveratrol and ε-viniferin on levels of proinflammatory (IL-6) and anti-inflammatory (IL-10) cytokines in the liver of rats with experimental severe acute liver failure using multiplex analysis. Values are expressed as the mean ± standard deviation. Lowercase letters indicate significant differences between treatments (*p* < 0.05), *n* = at least 4. RV: resveratrol + ε-viniferin; TAA: thioacetamide.

Figure 4 shows that the levels of the proinflammatory cytokine IL-6 were higher in the TAA group than in the control groups (CO and CO + RV; *p* < 0.05). The additional administration of the stilbenoids to the TAA-treated rats led to a significant reduction in the level of IL-6 activity in this group. The liver level of the anti-inflammatory cytokine IL-10 followed a parallel pattern, albeit at much lower intensities (Figure 4).

### 3.3. Assessment of mRNA Expression by Quantitative Real-Time PCR (RT-qPCR)

**Figure 5:**
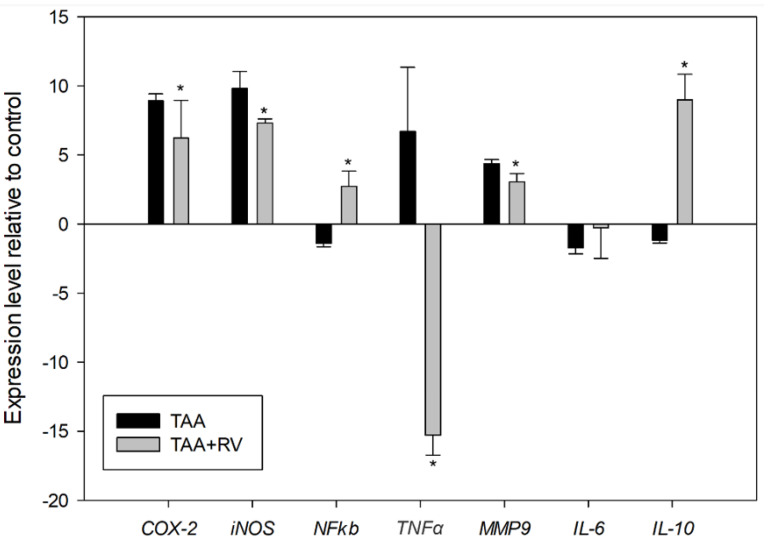
Transcriptional responses to the application of resveratrol and ε-viniferin in the liver of rats with experimental severe acute liver failure. Several genes related to inflammation and oxidative stress were analyzed. Values are expressed as the mean ± standard deviation. * Indicates significant differences between treatments (*p* < 0.05) (*n* = 6).

One way to assess the effects of resveratrol and ε-viniferin on TAA-treated rats was to determine the transcription of related genes. Figure 5 shows the expression of important inflammatory genes following the administration of resveratrol and ε-viniferin to TAA-treated rats. The transcription of the nuclear factor *NFκB* showed an upregulation relative to the control. The mRNA expression of the proinflammatory cytokines TNFα genes increased, and *IL-6* expression was not altered in the animals that received TAA as a hepatotoxicant when compared to the control. Administration of stilbenoids reversed this state, as they reduced the mRNA expression of *TNFα* compared to the TAA group, with a more severe downregulation of this gene compared to the control. Conversely, the administration of resveratrol and ε-viniferin did not affect the mRNA expression of *IL-6*. On the other hand, the mRNA expression of *IL-10* was upregulated when compared to the control and TAA groups. Other mediators of inflammation include iNOS and COX-2. In this study, *iNOS* and *COX-2* showed an upregulation of TAA and TAA + RV relative to the control, but resveratrol and ε-viniferin reduced the mRNA expression of these genes compared to TAA-treated animals. The administration of resveratrol and ε-viniferin also reduced the mRNA expression of *MMP-9* compared to the TAA-treated animals (Figure 5).

Data from a comet assay revealed a significant increase in DNA damage in TAA-treated rats. Resveratrol and ε-viniferin significantly reduced the DNA damage caused by TAA in the liver, as observed by the reduction in the damage index or damage frequency (Table 1).

## 4. Discussion

SALF syndrome causes a noticeable decline of hepatic function, leading to multiple organ failure, which is related to a high mortality rate. The cause of this disease remains unknown. The therapeutics available to avoid or treat SALF are still especially limited [8]. The use of TAA as a promoter of liver-tissue damage provided evidence that this xenobiotic leads to different degrees of liver injury in a dose-dependent manner. Hence, the aim of this study was to assess the potential protective effects of a combination of trans-resveratrol and trans-ε-viniferin against the damage caused by acute administration of TAA to rats. Although this model has been tested with resveratrol, to our knowledge, it has never been studied with viniferin or with both stilbenes combined. Two different processes were analyzed: the antioxidant pathway and selected chemical mediators of inflammation.

An increase in lipid peroxidation levels in the liver of animals undergoing TAA treatment was observed. In the stilbene-treated group, these increased levels were reduced, which might be explained by the combined antioxidant effects of resveratrol and ε-viniferin (Figure 2). Previous authors found comparable increases in TBARS in studies in which TAA was used to induce liver damage [27]. Resveratrol and ε-viniferin administration was able to reduce lipid peroxidation levels, in line with several previous studies with stilbenoids that used isolated compounds [28].

However, what could be the cause underneath this observed reduction in oxidative stress? CAT and SOD represented the first line of antioxidant defenses against oxidative damage. In our work, CAT liver activity decreased in the group in which TAA was administered in relation to both control groups. This decrease reflected the redox imbalance caused by stress resulting from TAA administration. Conversely, CAT activity increased in the group that received the combined resveratrol and ε-viniferin treatment, but its levels were still below those of the control groups (Figure 3B). Other studies using TAA reported similar results [10,29]. Studies using resveratrol as an antioxidant, but in different models, also reported identical results [30]. As in other studies using flavonoids [17] and vitamin E [27], the administration of resveratrol and ε-viniferin reduced the activity of SOD compared to TAA-treated rats, achieving values similar to the controls, possibly in an attempt to adjust for the liver damage caused by the presence of the xenobiotic (Figure 3A).

GST is an important cellular detoxification enzyme that works by removing toxic metabolites. In this study, TAA decreased the activity of liver GST, while resveratrol and ε-viniferin increased its activity (Figure 3C), in line with previous studies using plant extract [31].

The link between oxidative stress and inflammation has been recognized in many diseases and pathological conditions, including those that affect the liver. Extensive research during the last decades has unveiled, at least in part, the mechanisms by which oxidative stress can cause chronic inflammation [32]. Some signaling molecules are stimulated by ROS and activate inflammatory mediators, including NFκB, which regulates the expression of inflammatory cytokines (such as IL-6 and TNFα) and other mediators of inflammation (such as iNOS and COX-2) [29].

In this study, liver *NFκB* mRNA expression was upregulated in the TAA + RV group in relation to the control (Figure 5). In the liver, a critical prosurvival mechanism involves NFκB’s ability to maintain antioxidant defenses by controlling the expression of several key ROS-scavenging proteins [33].

Trans-resveratrol and ε-viniferin reversed the inflammatory process, as demonstrated by a downregulation of *TNFα* and an upregulation of *IL-10* in liver tissue after their administration to TAA-treated rats (Figure 5). A prior study using a TAA model for liver injury revealed an increase in the levels of proinflammatory cytokines, such as TNFα, TAA-induced liver-damaged animals [29]. As described by Pham et al. [33], the antiapoptotic activity of NFκB involves suppressing the accumulation of ROS and controlling the activation of the c-Jun N-terminal kinase (JNK) cascade, and in this way, suppressing the activity of TNFα. The same mechanism may explain our results. The combination of trans-resveratrol and ε-viniferin may act at a multilevel way to reverse the damage caused by the administration of this xenobiotic, increasing the effect caused by the isolated compounds *per si.*

The NFκB pathway regulates COX-2 production. The mRNA expression of *COX-2* causes a production and accumulation of a high concentration of prostaglandins, leading to inflammatory pathology. Meanwhile, iNOS induces the production of large amounts of nitric oxide, which displays free radicals and has been suggested to be an important inflammatory mediator [34]. In this study, *COX-2* mRNA expression increased in the rat livers of the group injected with TAA and decreased in the group that received TAA followed by ε-viniferin and resveratrol administration. These results (Figure 5) were in line with other studies in which TAA induced hepatotoxicity, The use of allopurinol increased *COX-2* mRNA expression, followed by decreased expression after treatment [35], similar to glutamine [7] and proanthocyanidin [36]. As a hepatotoxicant, TAA may stimulate the production of iNOS, and consequently NO synthesis, which leads to an increase inflammation in the liver. In this study, we observed overexpression of *iNOS* in the liver of animals with TAA-induced SALF. Stilbene administration was able to reverse these parameters, reducing *iNOS* mRNA expression (Figure 5). In a study using the intestinal ischemia–reperfusion model [9], and also hepatic fibrosis that was induced by TAA [34], a similar pattern of *iNOS* expression in the liver of treated animals was observed. These results were also in agreement with other results from a different model of liver inflammation using resveratrol. As in this work, an increased mRNA expression of *IL-10* and a suppression of *iNOS* and *TNFα* were observed [37].

In an attempt to shed light on the mechanisms involved in the cell-matrix degradation (ECM), we also evaluated the mRNA expression of *MMP-9*. It is known that extracts containing plant secondary metabolites can be associated with a reduction in fibrosis [38]. We demonstrated that the administration of resveratrol and ε-viniferin also exerted hepatoprotective effects against TAA damage, possibly via downregulation of *MMP-9* mRNA expression, leading to an inhibition of cell-matrix degradation caused by TAA (Figure 5). The downregulation observed in *MMP-9* mRNA expression may be related to the suppression of TNFα, as observed by Robert et al. [39]. The influence of stilbenes on *MMP-9* mRNA expression on an in vivo hepatic model is not well studied. Yu et al. [40] observed a reduction in MMP-9 activity caused by resveratrol in an in vitro model, also in a TNFα-dependent way. In the present work, however, the results suggested that the resveratrol and ε-viniferin combination may be more effective in reducing the MMP-9 than the isolated compounds.

Besides leading to lipoperoxidation, ROS may also induce oxidation of DNA [25]. Oxidative DNA damage is a common risk to genomic stability. In this study, increased DNA damage in liver tissues of the TAA-treated group was observed, suggesting an increase in genomic instability. These effects most likely were induced by ROS. Conversely, the animals in the TAA + RV group exhibited a reduction in liver DNA damage, suggesting a protective effect conferred by the ε-viniferin and resveratrol treatment (Table I). Other studies using a comet assay confirmed the protective role exhibited by resveratrol as a ROS scavenger, by decreasing DNA strand breaks and base oxidations [41].

It is known that resveratrol has high absorption but very low bioavailability in humans; 70% of the oral dose is absorbed, but its bioavailability is very poor, on the order of 5 ng/mL in the plasma. This was mainly due to rapid metabolism [42]. In rats, stilbene availability is also low [43]. Despite the low bioavailability, we were able to confirm that the resveratrol and ε-viniferin combination managed to reverse the adverse effects of TAA administration on the liver. For pharmaceutical researchers, understanding the concept of the extrapolation of doses between species is very important. Using the same dose applied in this study and making used of the equation described by Nair and Jacob [44], the estimated human equivalent dose is 1 mg/kg of each stilbene.

As a whole, our results demonstrated the hepatoprotective effects of a combination of trans-resveratrol and ε-viniferin, mostly through a protective role on the antioxidant pathway, via alteration on the activity of several important antioxidant enzymes (SOD, CAT, and GST); and on inflammation, activated by the downregulation of *TNFα* and upregulation of the *IL-10* pathway, and also *COX-2* and *iNOS*, in rats treated with this stilbene combination. We also showed evidence that the hepatic matrix was also protected by trans-resveratrol and ε-viniferin via downregulation of the *MMP-9* mRNA expression in the liver, which in turn (due to the strong link between MMP-9 activity, inflammation, and cancer) strongly suggested a reduction in the inflammatory process, and allowed us to infer a possible anticancer potential for this combination as well. All these observations make the combination of resveratrol and ε-viniferin a promising agent for reducing hepatocellular damage and treating liver toxicity.

In the current worldwide situation, when we observe an increase in the frequency of high-mortality-rated liver diseases, especially liver fibrosis, inflammation, and cancer, the finding of novel ways to deliver molecules with nutraceuticals properties that can improve or prevent these pathologies can be of significant importance.

## Figures and Tables

**Table 1:** Comet assay of the blood of rats with experimental severe acute liver failure and treated with resveratrol and ε-viniferin. Values are expressed as the mean ± standard deviation. Lowercase letters indicate significant differences between treatments (*p* < 0.05), *n* = 100 for each animal (7 animals per group). Co: control; RV: resveratrol + ε-viniferin; TAA: thioacetamide.

Group	Damage Index (0–400)	Damage Frequency (%)
CO	69.57 ± 9.79 ^c^	38.57 ± 4.03 ^c^
CO + RV	81.57 ± 22.58 ^c^	42.14 ± 9.90 ^c^
TAA	297 ± 15.16 ^a^	91.75 ± 3.40 ^a^
TAA + RV	176 ± 32.04 ^b^	71.66 ± 13.57 ^b^

## Data Availability

The data presented in this study are available upon request from the corresponding author upon reasonable request.

## Supplementary Materials

The following are available online at https://www.mdpi.com/article/10.3390/nu13113677/s1, Figure S1. Study design. Table S1: A list of the primers used for RT-qPCR.
