# Supplementary material for: Combination of Trans-Resveratrol and ε-Viniferin Induces a Hepatoprotective Effect in Rats with Severe Acute Liver Failure via Reduction of Oxidative Stress and MMP-9 Expression
Source: Nutrients. 2021 Oct 20;13(11):3677. doi: 10.3390/nu13113677 (PMC8622851; doi:10.3390/nu13113677)
Supplement: Supplementary file 1 [file nutrients-13-03677-s001.zip › nutrients-1408454-supplementary.pdf]

Supporting Information

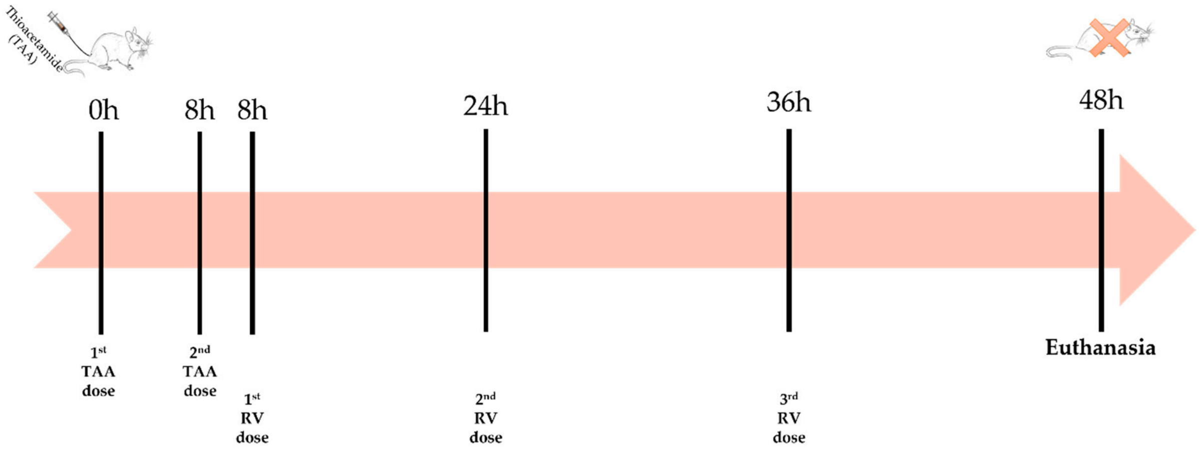

Figure S1. Study design.

Table S1. List of primers used for RT-qPCR

Accession number (NCBI)	ID attributed[1]	Primer sequence	Product length
NM_017232	RnCOX-2F	GAT TGA CAG CCC ACC AAC TT	149
	RnCOX-2R	CGG GAT GAA CTC TCT CCT CA	
XM_039085203	RniNOSF	GGA GAG ATT TTT TCA CGA CAC CC	74
	RniNOSR	CCA TGC ATA ATT TGG ACT TGC A	
XM_008772775	RnNFkBF	GAA AGT AAG GAT GTG GGA GGA G	156
	RnNFkBR	GTG GAT GAT GGC TAA GTG TAG G	
XM_008772775	RnTNFαF	ACT GAA CTT CGG GGT GAT TG	128
	RnTNFαR	GCT TGG TGG TTT GCTACG AC	
NM_031055	RnMMP9F	GTA ACC CTG GTC ACC GGA CTT	117
	RnMMP9R	ATA CGT TCC CGG CTG ATC AG	
NM_012589	RnIL-6F	TGT ATG AAC AGC GAT GAT GCA C	116
	RnIL-6R	ACG GAA CTC CAG AAG ACC AGA G	
XM_00624971	RnIL-10F	CTG CTA TGT TGC CTG CTC TTA	85
	RnIL-10R	GGG AAG TGG GTG CAG TTA TT	
NM_001394060	RnGAPDHF	GTA TTG GGC GCC TGG TCA CC	185
	RnGAPDHR	CGC TCC TGG AAG ATG GTG ATG G	
